# Ergot as an Anti-Galactagogue

**Published:** 1875-11

**Authors:** 


### ERGOT AS AN ANTI-GALACTAGOGUE.

A Russian physician, Dr. J. Schtscherbinenkoff, has made the interesting observation (*Centralblatt fur Chirurgie*, May 8, 1875,) during an epidemic, of secale cornutum in the district of Simbirski, that amongst the symptoms of ergot-poisoning is the diminution or complete arrest of the secretion of milk in lactating women. The same result was found to occur in cows that had been fed on meal which contained ergot, or that had been littered with carelessly-threshed straw which still contained some affected ears. Schtscherbinenkoff conceived the idea of employing it as a remedy in cases of threatened abscess of the breast, and carried it out in many cases with great advantage. Two multiparæ, who had suffered at each previous confinement from abscess of the breast, took some of the drug with the happiest result, as soon as swelling began to appear, accompanied by congestion of the gland with milk. He has also administered ergot and quinine, in doses of three grammes of each, twice or thrice a day, in cases of so-called milk-fever, and has found the drug useful in cases of swelling of the breast, accompanied by fever, in other than puerperal women, as well as in those where it is required to wean the child either at the normal or at an earlier period. He has exhibited as much as a drachm of ergot daily, for a week, without observing any unpleasant result.—*Western Lancet.*

